# Policy by Pilot? Learning From Demonstration Projects for Integrated Care: Comment on "Integration or Fragmentation of Health Care? Examining Policies and Politics in a Belgian Case Study"

**DOI:** 10.34172/ijhpm.2022.7152

**Published:** 2022-06-08

**Authors:** Radhika Gore

**Affiliations:** Family Health Centers at NYU Langone, Brooklyn, NY, USA.

**Keywords:** Chronic Disease, Social Determinants of Health, Health Equity, Health Service Delivery, Social Services, Policy Learning

## Abstract

Analysis of policy implementation for chronic disease in Belgium highlights the difficulties of launching experiments for integrated care in a health system with fragmented governance. It also entreats us to consider the inherent challenges of piloting integrated care for chronic disease. Sociomedical characteristics of chronic disease –political, social, and economic aspects of improving outcomes – pose distinct problems for pilot projects, particularly because addressing health inequity requires collaboration across health and social sectors and a long-term, life-course perspective on health. Drawing on recent US experience with demonstration projects for health service delivery reform and on chronic disease research, I discuss constraints of and lessons from pilot projects. The policy learning from pilots lies beyond their technical evaluative yield. Pilot projects can evince political and social challenges to achieving integrated chronic disease care, and can illuminate overlooked perspectives, such as those of community-based organizations (CBOs), thereby potentially extending the terms of policy debate.

 Barriers to integrated care are not technical; they are political,” wrote Berwick et al in 2008, referring to the challenges of instituting the Triple Aim of improving individual care, enhancing population health, and reducing cost of care.^1^ Integrated care, as defined in their paper, is conditioned partly on the existence of an “integrator,” an organization responsible for all three aims for an identified population. Martens and colleagues’ study of policy implementation for chronic disease in Belgium, specifically their analysis of 12 pilot projects, highlights the difficulties of launching experiments to model pathways to integrated care in a health system without a strong integrative force.^2^

 Research covering the pilot projects’ initial stages suggested their iterative, collaborative process had prompted new interdependencies and a nascent “negotiated governance” among pilot project stakeholders and authorities.^3^ Martens and colleagues show how shifts in conditions, such as the federal government’s favor of short-term efficiencies and its undoing of the legal basis to redistribute responsibilities between federal and federated levels, left pilot project participants without incentives to collaborate towards long-term gains and without the ability to finance activities at federated level.^2^ The pilots gained little traction in these conditions. The analysis illuminates how a fragmented governance structure and divided responsibilities are diametric to the goals of integrated care. It also entreats us to consider the inherent challenges of piloting integrated care for chronic disease.

 As Martens and colleagues describe, the projects were instituted as part of an ambitious plan with a bottom-up strategy to develop regional pilots that would generate lessons to reform chronic disease care and financing and improve health equity.^2^ These goals depend on sustained political commitment and concerted, long-term multisectoral action. To what extent are pilot projects, which are typically time-limited experiments, suited for this aim? What technical and political lessons may we learn from pilots for integrated care?

## Chronic Disease and Pilot Projects

 Governments are known to use pilot projects as a policy instrument precisely when fragmented state institutions and partisan divisions make it difficult to introduce large-scale reform.^4^ Policymakers have turned to analytic pilot projects to anticipate the potential effects of reform proposals before legislating policy. Pilot projects can allow governments to try new strategies, innovate, and gain public and political support for a policy idea. They can also be used as policy implementation tools: they can be structured as experiments to allow stakeholders to interact, exchange perspectives and knowledge, and develop new solutions for policy implementation in a staged manner, with room for trial and error.^3^

 However, the sociomedical characteristics of chronic disease – the political, social, and economic aspects of improving outcomes – pose distinct problems for pilot projects. Preventing disease, promoting health, enhancing self-management of chronic conditions, and reducing inequalities in outcomes requires addressing the social determinants of health. This entails collective action among diverse entities across health and social sectors; payment mechanisms that reflect the contributions of entities in each sector; concerted patient engagement and community outreach, which in turn relies on the nature of social ties between clinics and the communities they serve; and sufficient time to demonstrate health impacts from ameliorating social risks.

 I discuss these points in turn, reflecting especially on the United States’ experience. Since the 1960s, policymakers in the United States have increasingly used demonstration projects to test policy proposals. Demonstration projects featured prominently under the 2010 Patient Protection and Affordable Care Act (ACA).^4^ The ACA presented imperatives for healthcare organizations to achieve the Triple Aim and included a focus on chronic disease. For example, the ACA encouraged healthcare systems to integrate population-health perspectives in medical care, design service delivery to align with value-based payment, assess and respond to community needs, and invest in disease prevention.^5^ Along with lessons from demonstration projects, I draw upon research on chronic disease as it bears upon the question of the pilot project as a useful policy instrument. While the evidence covered here focuses on chronic diseases such as hypertension and diabetes, the question is relevant generally for integrated health and long-term social care, which aims to integrate social care systems across the life course.^6^

 Tackling the connection between social determinants of health and chronic disease calls for multisectoral action. Integrated care for chronic disease entails not only coordinating care at the point of delivery, but rethinking and reforming how healthcare, welfare, and other systems operate together.^7^ It involves managing immediate priorities of different systems as well as grappling with the institutional histories, practices, and norms that define their structures and workings. The “institutional arrangements that make up a polity” emerge from various processes forged at different times and may not cohere in their operations or functions.^8^ As Minkman writes of the Netherlands experience, even when networks for collaboration are agreed upon and instituted, collaborative action is “not automatic in practice.”^7^ Powerful organizations can dominate collective efforts. Organizations may differ in their values. Governance within organizations may not align with modes of decision-making, accountability, and trust involved in the governance of inter-organizational networks. These dissimilarities can compound the challenges of overcoming political fragmentation in a federal system, particularly within a pilot project timeframe.

 Multisectoral action additionally foregrounds the “wrong pocket” problem, where costs for prevention may be borne by one entity (eg, investing in education, parks, transport) but rewards accrue to another (eg, saving in medical costs), possibly at another governance level.^9^ Even with flexibility to structure financing, this presents a technical and political tangle for integrated care: identifying the contributions of diverse entities and accounting for the economic cost of chronic disease. It is possible to conceptualize the economic case for treating a chronic disease such as hypertension at scale, namely that treating hypertension achieves long-term savings in medical costs and provides a net return to society. However, evidence on the economics of chronic disease is limited and uncertain and depends on the data, assumptions, and analytic methods used.^10,11^ Different kinds of preventive care, such as screening individuals for hypertension versus community-wide physical activity promotion and salt reduction, may be performed by different entities and have interrelated effects.

 Within healthcare, addressing patients’ social conditions involves connecting clinical and social services and responding to individual social risks at point of care. It requires proactively reaching people, such as for screening and education to prevent disease, and ensuring patients regularly connect with providers to manage disease. Fostering patient engagement in turn relies on the nature and strength of social ties between clinics and the communities they serve.^12^ Such relationships are typically developed through long-term deliberation and consultation. Policy implementation experience suggests how local social contexts can complicate building ties. The ACA mandated nonprofit hospitals to conduct community health needs assessments, expanding their community-based obligations as part of maintaining their tax-exempt status. But hospitals and communities can hold differing perceptions about shared neighborhood space and definitions of “community.”^13^ Differences in perspectives can “lead to disjunctions in developmental planning and health-related community programming.”^13^ Pilot projects to address the social determinants of chronic disease may confront such tensions, which require time and relationship-building to resolve.

 Time horizons additionally matter to obtain health effects from integrating health and social care. For patients, resolving social needs, managing disease, and sustaining healthy behavior are lengthy, complex, intertwined processes. Stresses and strains can accrue over the life course, and transitions from adolescence to adulthood may be associated with different pathways and implications for health than later life course transitions.^14^ Evaluations of short-term pilots may be unable to fully show effectiveness when meaningful impacts occur over years or generations, and would need to account for differential needs and impacts across the life course.^14^

## Learning From Pilot Projects

 What can pilot projects teach us about integrated care? The Belgian case suggests pilots can surface implementation challenges, specifically collective action challenges, before they are able to show effectiveness. As experiments in collaboration, pilot projects can lay bare power differentials among health system actors. An example of a demonstration program that has yielded insights into local partnership and financing dynamics is the Delivery System Reform Incentive Payment (DSRIP) program in the United States. DSRIP is a federal program to test strategies to achieve the Triple Aim for the population covered by Medicaid, the public health coverage program for low-income adults and children. To receive DSRIP funds, states must propose a plan to reform health service delivery systems towards integrated care and improved outcomes for Medicaid beneficiaries, with payment tied explicitly to outcomes.^15^ DSRIP incentivized healthcare systems to incorporate support for social services, and in the process uncovered the challenges of doing so.

 New York State began its five-year DSRIP program in 2014. The state’s Department of Health formed 25 delivery networks known as performing provider systems (PPS), each led by a healthcare entity and comprising hospitals, primary care and specialist physicians, and social service providers, among others.^15^ Each PPS was required to form a governing body to oversee planning and implementation of projects they could select from a menu of options; the options included chronic disease-related goals. PPSs had to “determine how to distribute DSRIP funds, support the use of health information technology, develop patient and provider engagement strategies, and be accountable to the state.”^15^

 PPS governance both reflected and influenced relationships among partnering organizations. PPS networks included numerous community-based organizations (CBOs), but some CBOs played significant roles in governance processes while others engaged little. As Felland and colleagues note, for healthcare entities leading the PPSs, contracting with CBOs was complex and time-consuming where their services were not reimbursable through Medicaid or DSRIP funds were not known in advance.^15^ Due to their limited capacity and resources, CBOs could not easily contribute effort without upfront funding. There were also tensions in allocating funds between health and social service providers. PPS leaders found it difficult to link nonmedical activities to clinical process measures. As DSRIP milestones shifted from process to performance measures (requiring a more comprehensive approach) and as CBOs advocated for their position, fund flows shifted.^15^ Yet CBOs expressed that value-based payment models were not structured to include them and questioned the sustainability of their efforts.^16^

 Unresolved questions about social service reimbursement have spurred scholarship on clinic-community linkages and CBOs’ perspectives on Medicaid redesign. Among the issues highlighted are a disjuncture in mission and interests between CBOs and healthcare providers, CBOs’ enacting organizational changes to gain legitimacy with healthcare partners (eg, hiring clinical staff to management positions) to benefit from resources flowing from healthcare entities, and CBOs’ concerns about medicalizing social care.^17^ DSRIP not only pointed to the implementation challenges of integrating medical and nonmedical services, but also instigated social-scientific analysis of intersectoral power, inter-organizational relationships, and the potential medicalization of social service assistance. Pilot projects can, in this way, introduce new policy-relevant debates on chronic disease. If politics are the main barrier to integrated care, where integration includes multisector collaboration to address the social determinants of health, then pilot projects may not only reveal where political fault lines lie but also generate opportunities to clarify and articulate social issues.^18^

## Conclusion

 Pilot projects with short time horizons seem unsuited to demonstrate improvements in chronic disease outcomes and health equity. But the policy learning from pilots extends beyond their technical evaluative yield. Pilot projects can evince political and social challenges to achieving integrated chronic disease care, and can illuminate overlooked perspectives, such as those of CBOs, potentially extending the terms of policy debate.

## Ethical issues

 Not applicable.

## Competing interests

 Author declares that she has no competing interests.

## Author’s contribution

 RG is the single author of the paper.

## Disclaimer

 The views and opinions expressed in this commentary are mine alone, and do not necessarily reflect or represent those of the Family Health Centers at NYU Langone.
